# La dissection spontanée des artères coronaires: à propos de 2 cas

**DOI:** 10.11604/pamj.2017.28.164.9870

**Published:** 2017-10-20

**Authors:** Abdelouahab Elkasimi, Ghizlane Elouazzani, Anas Hbali, Nabila Ismaili, Nouha Elouafi

**Affiliations:** 1 Service de Cardiologie, CHU Mohammed VI, Faculté de Médecine et de Pharmacie d’Oujda, Université Mohamed Premier, Maroc

**Keywords:** Dissection coronaire spontanée, syndrome coronarien aigu, athérosclérose, Spontaneous coronary dissection, acute coronary syndrome, atherosclerosis

## Abstract

La dissection spontanée des artères coronaires (DSAC) est une cause rare du syndrome coronarien aigu (SAC); sa prévalence est de moins de 3%. Elle se définit par une séparation au sein de la paroi artérielle coronaire secondaire à une hémorragie intra murale, avec ou sans déchirure de l’intima, créant un faux chenal. Elle survient essentiellement chez les sujets jeunes, principalement les femmes, sans athérosclérose coronarienne ni facteurs de risque d’athérosclérose; toutefois la survenue d’une dissection spontanée sur une maladie athéromateuse est possible. A travers 2 cas de dissection coronaire spontanée, nous discutons sa pathogénie, les facteurs favorisant sa survenue, sa présentation clinique et sa gestion thérapeutique.

## Abstract (en)

Spontaneous coronary artery dissection (SCAD) is a rare cause of acute coronary syndrome (ACS); its prevalence is estimated to be less than 3%. It is defined as a separation within the coronary arterial wall secondary to intramural bleeding with or without intimal tear, creating a false channel. It mainly affects young subjects, mainly women, without coronary atherosclerosis or risk factors for atherosclerosis. However, the occurrence of spontaneous dissection due to atheromatous disease is possible. We here report 2 cases of spontaneous coronary dissection, in order to discuss its pathogenesis, factors favoring its occurrence, its clinical presentation and its therapeutic management.

## Introduction

La dissection spontanée de l’artère coronaire(DSAC) est une cause rare du syndrome coronarien aigu (SCA), qui survient essentiellement chez les sujets jeunes, principalement les femmes, sans athérosclérose coronarienne ni facteurs de risque d’athérosclérose [1]. Sa prévalence est de moins de 3% [2] et elle est responsable de 10% à 30% d’infarctus myocardique chez les femmes de moins de 50 ansessentiellement au cours de la période du post-partum [3-5]. Malgré que la DSACpeut être un événement fatal, la plupart des séries suggèrent que la survie précoce et à long terme est bonne [1,6], cependant, la survenue de complications est notable. Les facteurs déclenchant la dissection coronarienne sont incertains, l’absence de facteurs de risque typiques, et l’aspect des vaisseaux sur l’imagerie intra vasculaire suggèrent que le mécanisme physiopathologique est différent du mécanisme ischémique atherothrombotique dans l’infarctus du myocarde (IM) [1,7,8]. Par conséquent, la stratégie de gestion à court et à long terme diffère également. A travers 2 cas de dissection coronaire spontanée, nous discutons sa pathogénie, les facteurs déclenchants, les indications thérapeutiques ainsi que la particularité de notre série.

## Patient et observation

**Figure 1:**
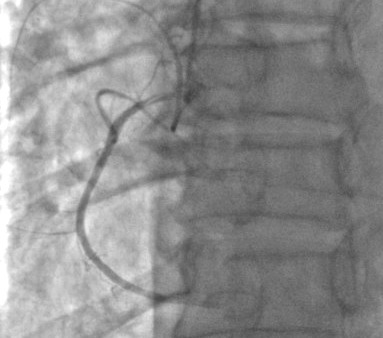
Dissection du 1^er^ segment de la coronaire droite

**Figure 2:**
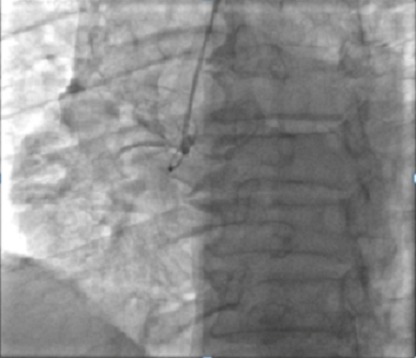
Occlusion de la coronaire droite en aval de la dissection

**Figure 3:**
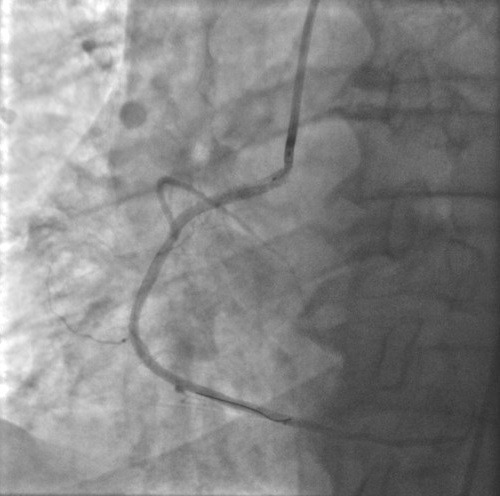
Recanalisation de la coronaire droite après un traitement antiagrégant intensif et une angioplastie

**Observations n°1:** il s’agit d’un patient âgé de 67 ans, admis pour prise en charge d’un SCA avec sus décalage en inférieur en rapport avec une dissection spontanée du 1^er^ segment de la coronaire droite (Figure 1) suivie rapidement d’une occlusion située juste en en aval de la dissection (Figure 2), vu l’état hémodynamique stable du patient et vu l’absence d’un centre de chirurgie cardiovasculaire dans notre formation, celui-ci a été mis sous traitement médical à base d’ antiagrégants plaquettaires incluant l’Aspirine, le Clopidogrel et les anti GP IIb/IIIa (AGRASTAT) associé au reste du traitement anti angineux classique. Le contrôle angiographique a montré une recanalisation spontanée de la coronaire droite avec mise en évidence d’une sténose serrée ostiale ayant fait l’objet d’une angioplastie avec mise en place d’un stent actif et l’évolutionà court et à moyen terme était bonne (Figure 3).

**Figure 4:**
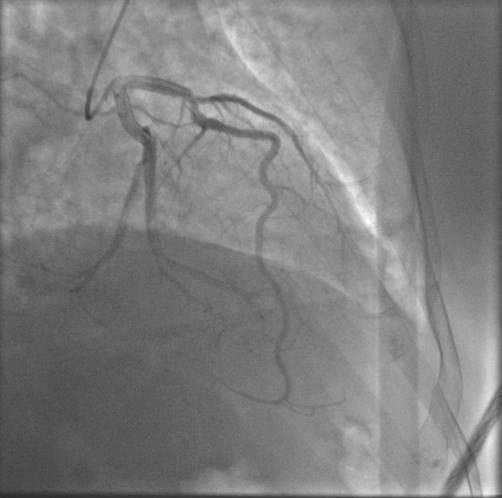
Dissection de l’IVA allant depuis son ostium jusqu’à sa partie moyenne

**Observation n°2:** il s’agit d’un patient âgé de 76 ans, admis aux urgences pour prise en charge d’un SCA ST positif en antérieur thrombolysé à H7 de la douleur. Devant la persistance du sus décalage une coronarographie a été réalisée objectivant une dissection de l’IVA depuis son ostium allant jusqu’à sa partie moyenne avec une sténose intermédiaire de la bifurcation (Figure 4). Le patient a été traité médicalement avec une bonne évolution.

## Discussion

La dissection spontanée de l’artère coronaire est une cause rare du syndrome coronarien aigu et de mort subite dont l’étiopathogénie est toujours mal élucidée, affecte préférentiellement la population en bonne santé et jeune surtout de sexe féminin. Elle se définit par une séparation au sein de la paroi artérielle coronaire secondaire à une hémorragie intra murale, avec ou sans déchirure de l’intima, créant un faux chenal. Cette séparation peut siéger entre l’intima et le média ou entre le média et l’adventice [9]. Elle a été découverte lors d’autopsie de mort subite et sa première description a été rapportée en 1931 par Pretty [10]. Sa physiopathologie n’est pas encore complètement élucidée mais il existe des facteurs bien connus qui peuvent induire une faiblesse du collagène et prédisposer à la dissection coronaire . Cette faiblesse peut être acquise comme lors d’une grossesse ou d’une contraception orale, mais aussi congénitale comme lors de maladie de Marfan ou de syndrome d’Ehlers-Danlos [11, 12]. L’hypothèse d’une action cytotoxique et collagénolytique des éosinophiles présents dans l’adventice en postpartum a été retenue mais on identifie égalementl’origine médicamenteuse (cocaïne, ciclosporine); le traumatisme thoracique et le stress physique ou psychique comme facteurs déclenchant la dissection coronarienne [13, 14] .

Toutefois la survenue d’une dissection spontanée sur une maladie athéromateuse est possible, ceci est bien illustré dans les observations des 2 patients de notre série qui sont de sexe masculin; âgés de plus de 50 ans et ayant tous les deux des facteurs de risque de maladie coronarienne avec une exploration coronarographique confirmant l’origine athéromateuse de la dissection coronarienne spontanée. La présentation clinique de la DSAC est celle d’un SCA avec ou sans élévation du segment ST, elle peut se présenterégalement comme un angor instable; une dyspnée ou une mort subite [1,6]. Le diagnostic positif comporte un électrocardiogramme anormal, des biomarqueurs cardiaques élevés et des troubles de la cinétique sur l’échocardiographie. Étant donné que ces patients sont souvent jeunes et en bonne santé, le SCA peut ne pas être évoqué comme diagnostic initial; cette absence de suspicion de SCA chez les patients avec DSACpeut empêcher ou retarder le diagnostic approprié si l’électrocardiogramme et l’élévation de troponine ne sont pas inclus dans l’évaluation initiale. Les patients peuvent plutôt être diagnostiqués à tort, comme ayant une péricardite, une cardiomyopathie de Takotsubo, ou un reflux gastro-intestinal sansbilan approprié pour SCA; d’où l’intérêt de suspecter et d’éliminer de façon appropriée une ischémie aigue. Lorsque le SCA est reconnu, l’angiographie coronarienne doit être effectuée immédiatement. Sur la coronarographie, la DSACpeut apparaître comme une double lumière en raison de remplissage du contraste dans un plan de dissection intimale (faux lumière). Cependant, la DSAC se présente couramment avec un hématome intramural sans flap intimal visible. Ce qui peut mimer l’aspect d’un vaso-spasme, d’athérosclérose, ou de coronaires normales. Par conséquent, dans ces cas, l’échographie intravasculaire(EIV) ou la tomographie par cohérence optique (OCT) au moment de l’angiographie devraient être utilisées pour diagnostiquer l’hématome intramural secondaire à la DSAC [15].

**Figure 5:**
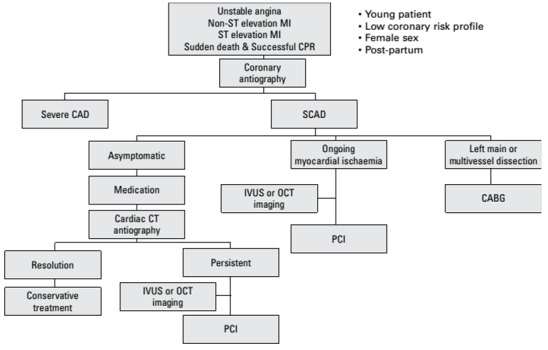
Algorithme pour le diagnostic et le traitement de la dissection coronaire spontanée

L’OCT a une résolution spéciale de 10-20 mm et peut clairement différencier l’hématome intra-murale (HIM) des plaques lipidiques ou des lésions calcifiés elle différencie également la vrai et la fausse lumière, et permet de visualiser les déchirures de l’intima. l’EIV a une résolution spatiale basse (150 mm), cependant elle permet de visualiser les déchirures de l’intima, la vrai et la fausse lumière, ainsi l’ HIM apparaît comme collection homogène derrière la membrane intimale-médiale [16]. Sur le plan thérapeutique la gestion à court terme des patients présentant uneDSAC diffèrent des recommandations du SCA sur maladie athéroscléreuse [1,17], car ces patients ont des taux élevés de complications secondaires aux interventions coronaires percutanées (ICP), même chez ceux qui présentent un flux sanguin coronaire préservé [1,6,17]. Les thrombolytiques devraient être évité dans la DSAC vu le risque accru d’une extension de la dissection. Dans une étude rétrospective de Shamboo et al [18], 60% des patients thrombolysés ont nécessité le recours à une intervention coronaire percutanée ou une chirurgie de sauvetage.Un patient de notre série a bénéficié d’une thrombolyseen urgence sans signes de succès mais également sans complications. La décision thérapeutique dépend de l’état hémodynamique du patient et de la localisation de la lésion (Figure 5) [19]. Pour les patients présentant un arrêt du débit sanguin coronaire ou une instabilité hémodynamique, une PCI ou un pontage aorto-coronaire peuvent être proposé afin de sauver le myocarde viable, bien que le taux d’échec de la procédure de PCI peut être aussi élevé [17]. Dans notre série la technique de PCI était utilisée chez un seul patient après un traitement médical initial avec des suites simples et une bonne évolution à long terme. Chez les patients qui sont par ailleurs stables avec un flux sanguin coronaire préservé, la gestion conservatrice et une surveillance étroite sont associées à des résultats favorables [1,6,17,18]. Le traitement médical associeun antiplaquettaire unique, un bétabloquant et un traitement par statine pour les patients avec une dyslipidémie, ceci était réalisé chez nos deux patients. La surveillance doit se faire en milieu hospitalier pendant 5 jours car ces patients sont à risque d’événements futurs, y compris une DSACrécurrente, infarctus de myocarde et insuffisance cardiaque congestive [17]. Le traitement médical comporte un antiagrégant plaquettaire ou une double anti agrégation pour les patients ayant fait l’objet d’une angioplastie percutanée. Les statines ne sont pas systématiquement prescrites, mais recommandé pour ceux qui ont une hyperlipidémieou une origine athéromateuse. Les bétabloquants et les inhibiteurs de l’enzyme de conversion sont indiqués en cas dysfonctionnement ventriculaire gauche. Le rôle des inhibiteurs GPIIb / IIIa dans la PEC des DCSA n’est pas encore évalué, mais vu le risque de saignement qu’ils peuvent engendrer et le risque potentiel de faire étendre la dissection, ils ne sont pas utilisés en routine [16], les antis GPIIb / IIIa ont été utilisés chez l’un de nos patientsavec bonne évolution. Chez les femmes en âge de procréer; les contraceptifs oraux sont à éviter et à remplacer parune contraception mécanique voire une ligature des trompes chez les femmes en âge de procréer. En raison de la prévalence notable des anomalies artérielles extra-coronaire associées à la dissection coronaire, il est conseillé de compléter par une imagerie du système vasculaire. Un angioscanner thoracique a été réalisé chez un de nos patients à la recherche d’une dissection aortique mais revenant normal.

## Conclusion

La dissection coronaire spontanée est l’une des causes de mort subite d’origine cardiaque et de SCA, en particulier chez les jeunes femmes; cependant elle peut se voir également chez le sujet âgé chez qui l’origine athéromateuse doit être évoquée en première intention afin de prodiguer une meilleure prise en charge thérapeutique.

## Conflits d’intérêts

Les auteurs ne déclarent aucun conflit d’intérêts.
